# Protective Effect of B1 and B12 Vitamins on Post‐Thaw Sperm Quality Parameters and Seminal Plasma Antioxidant Status in Rams

**DOI:** 10.1002/vms3.70241

**Published:** 2025-02-06

**Authors:** Saleh Tabatabaei Vakili, Roghayeh Zeidi, Robab Nabhani

**Affiliations:** ^1^ Department of Animal Science Faculty of Animal Science and Food Technology Agricultural Sciences and Natural Resources University of Khuzestan Mollasani Iran

**Keywords:** cobalamin, cryopreservation, ram, semen quality, thiamine

## Abstract

**Background:**

Sperm membranes, rich in polyunsaturated fatty acids, are highly susceptible to lipid peroxidation. Oxidative stress significantly impacts sperm function, and B‐group vitamins play a crucial role in mitigating this damage.

**Objective:**

This study investigated the effects of vitamins B1 (VitB1) and B12 (VitB12) on sperm quality and seminal antioxidant status following cryopreservation in Arabi rams.

**Methods:**

Semen was collected from 10 Arabi rams and pooled. The pooled semen was diluted and cryopreserved with varying concentrations of VitB1 and VitB12 (0, 0.5, 1, 2 and 4 mg/mL). Post‐thaw sperm quality parameters, including motility, viability, membrane integrity and morphology, were assessed. In addition, seminal antioxidant enzyme activities and lipid peroxidation were evaluated.

**Results:**

Supplementation with 1 mg/mL VitB12 and 0.5–1 mg/mL VitB1 significantly enhanced post‐thaw sperm motility, viability, membrane integrity and morphology. This was accompanied by a reduction in malondialdehyde (MDA) levels. The highest total antioxidant capacity (TAC) was observed with 1 mg/mL VitB12 and 0.5 mg/mL VitB1 (*p* < 0.05), positively correlating with sperm motility and viability (*p* < 0.01). Glutathione peroxidase (GPx) activity was highest in the 1 mg/mL VitB12 and VitB1 groups and negatively correlated with sperm abnormalities (*p* < 0.01). Catalase (CAT) activity significantly increased in the 1–2 mg/mL VitB12 and 0.5–2 mg/mL VitB1 groups, positively correlating with sperm progressive motility and viability (*p* < 0.01).

**Conclusions:**

These findings demonstrate that incorporating 1 mg/mL VitB12 and 0.5 mg/mL VitB1 into the cryopreservation extender effectively mitigates oxidative stress, enhances antioxidant defences and improves post‐thaw semen quality in Arabi rams.

## Graphical abstract

This study investigated the in vitro effects of B1 and B12 vitamins on sperm quality and seminal antioxidant activity in rams. The results showed that adding 1 mg/mL VitB12 or 0.5 mg/mL VitB1 to the diluent significantly improved ram sperm quality and antioxidant function in semen.

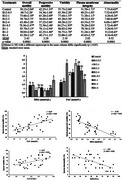

## 1 Introduction

The Arabi sheep, a breed indigenous to Iran, is well‐regarded for its dual‐purpose capabilities in both meat and wool production. This breed particularly excels in the humid tropical conditions of Khuzestan province (Roshanfekr et al. 2015). One major concern affecting sperm quality in these sheep is oxidative stress, which arises from an imbalance between oxidants and antioxidants and can impair sperm function (Giannattasio et al. 2002). Sheep sperm membranes are particularly susceptible to oxidative damage due to their high levels of polyunsaturated fatty acids (PUFAs). During storage, reactive oxygen species (ROS) can inflict significant damage (Y. Wang et al. 2022). Elevated levels of ROS, such as superoxide radicals, hydrogen peroxide (H_2_O_2_), hydroxyl radicals and lipid peroxides, can adversely affect cellular components, including proteins, membrane lipids and nucleic acids (Dabagh et al. 2023; C. Wang et al. 2006). These oxidative stress‐induced changes in the sperm membrane can lead to diminished sperm function and fertility. Moreover, the procedures of cooling, freezing and thawing sperm, particularly at subzero temperatures, add extra stress to the spermatozoa membrane (Jumintono et al. 2021). The cryopreservation process exacerbates oxidative stress, resulting in reduced sperm motility and viability (Daghigh Kia and Vatankhah 2019).

Seminal fluid contains a variety of antioxidants, both enzymatic and non‐enzymatic, which together enhance its total antioxidant capacity (TAC) (Cofré‐Narbona et al. 2016). Enzymatic antioxidants such as superoxide dismutase (SOD), glutathione peroxidase (GPx) and catalase (CAT) play a crucial role in neutralizing oxidative stress. In addition, non‐enzymatic antioxidants, including essential amino acids, vitamins and minerals, are critical for energy metabolism and the development of spermatozoa. These components help maintain sperm DNA integrity, potentially safeguarding it from oxidative damage (Patki et al. 2023).

B vitamins, especially B1 (thiamin) and B12 (cobalamin) are essential for cellular metabolism and development (Kennedy 2016). Insufficient levels of these vitamins have been linked to diminished semen quality and male infertility. Conversely, supplementation with B vitamins has enhanced sperm parameters, particularly during cryopreservation (Hassani‐Bafrani et al. 2019).

Vitamin B12 (VitB12) is essential for cellular metabolism, especially in one‐carbon metabolism, which is necessary for DNA synthesis and cellular function (Bärebring et al. 2023). VitB12 has a direct relationship with sperm motility and maintains mitochondrial function, which is crucial for energy production in sperm cells. Studies have shown that VitB12 deficiency can result in poor sperm motility and increased sperm abnormalities, whereas supplementation with VitB12 improves motility, morphology and overall sperm quality. This is likely due to its ability to reduce oxidative stress and enhance the activity of antioxidant enzymes like GPx, which protect sperm membranes from damage caused by ROS (Banihani 2017; Hu et al. 2011). In addition, VitB12 may help modulate the production of ROS in sperm, preventing the damaging effects of excessive oxidative stress during processes like cryopreservation. In studies involving male rats, a deficiency in VitB12 has been linked to decreased sperm motility and an increase in abnormal sperm. On the other hand, some research suggests that VitB12 supplementation can improve sperm motility and count (Hu et al. 2011).

However, an excess of VitB12 in semen extenders might counteract oxidative stress by promoting excessive ROS formation, which can adversely affect sperm functions related to ROS. Thus, selecting the appropriate concentration of antioxidants is important (El‐Darawany 1999; Hamedani et al. 2013).

Vitamin B1 (VitB1), or thiamine, plays a crucial role in carbohydrate metabolism and oxidative processes, contributing to energy production and cellular function. It acts as a coenzyme in the pentose phosphate pathway, which generates NADPH, the reducing agent required by antioxidant systems to neutralize ROS. In the context of sperm function, VitB1 helps maintain sperm motility and viability by supporting mitochondrial function and enhancing the sperm's ability to cope with oxidative stress (Hassan et al. 2020). Also, VitB1 has not been as widely studied concerning sperm quality as VitB12; its role in oxidative stress protection is becoming increasingly recognized. It helps prevent sperm membrane damage caused by ROS, improving sperm motility and membrane integrity, particularly during cryopreservation, when oxidative damage is most pronounced (Hassan et al. 2020).

While the antioxidant effects of VitB12 in semen have been explored in several species, the influence of VitB1 on male fertility is less well‐documented. The innovative aspect of this study lies in the individual use of VitB1 and VitB12 in cryopreservation extenders for rams. Although previous studies have examined the effects of VitB12 in various species, the specific impact of VitB12 supplementation on sperm quality and antioxidant status in rams after cryopreservation remains insufficiently explored. In addition, while VitB1's role in spermatogenesis and oxidative stress mitigation has been noted in other species, its potential to improve sperm quality during cryopreservation in rams is yet to be fully investigated. This study aims to address these gaps by assessing the effects of VitB12 and VitB1 separately in cryopreservation media, focusing on their impact on sperm motility, viability, membrane integrity and antioxidant activity.

## 2 Materials and Methods

### 2.1 Study Location and Animals

This study was conducted at the Agricultural Sciences and Natural Resources University of Khuzestan research farm in Khuzestan province, Iran (latitude 31°52ʹ N). The climate in this region is semi‐arid, characterized by hot summers and mild winters, with seasonal fluctuations in temperature and humidity. The breeding season for the Arabi rams in this region typically spans from October to December, while the non‐breeding season occurs from January to early March. A total of 10 adult Arabi rams, aged 3–4 years and with an average body weight of 60 kg (±2 kg), were selected for the study. These rams were selected based on their health status and reproductive history to ensure they were sexually mature and had no known reproductive issues. All animals were monitored for any signs of disease or stress throughout the study. The animals were kept in indoor group housing and fed according to National Research Council (NRC) recommendations. Their diet comprised alfalfa (40%), wheat straw (20%), corn (25%), wheat bran (13%), salt (0.5%) and a mineral supplement (0.5%). This diet provided a crude protein level of 15% and a metabolizable energy content of 2.3 Mcal/kg, ensuring adequate nutrition during the study. All rams had access to fresh water ad libitum.

### 2.2 Collection and Dilution of Semen

Semen was collected from each ram during the non‐breeding season (January to early March) using an electroejaculator (Ogawa Seiki Co. Ltd., Japan). Before collection, the rams were gently restrained. The electroejaculation procedure was conducted following standard guidelines to ensure the animals' welfare and minimize discomfort. Following collection, the ejaculates were promptly transported to the laboratory and incubated in a water bath at 37°C. The semen samples were immediately transferred to the laboratory in an insulated container to maintain the temperature at 37°C. Upon arrival at the laboratory, semen samples were incubated in a water bath at 37°C for 10 min to stabilize the sperm motility. Only samples with a minimum sperm concentration of 2 × 10^9^ sperm/mL, a motility rate greater than 80% and a volume between 1 and 2 mL were selected for use in the study. Samples were pooled from all rams to reduce individual variability in semen quality. The selected pooled semen was then diluted in a tris‐based extender. The extender was prepared by dissolving the following ingredients in distilled water: Tris (3.63 g), fructose (0.5 g), citric acid (1.99 g), egg yolk (14%), glycerol (5%), penicillin (100,000 IU) and streptomycin (100 mg). The final volume was adjusted to 100 mL with distilled water. The pH of the extender was adjusted to 7.2 using a pH meter. The semen was diluted in a 1:10 ratio, achieving a final sperm concentration of approximately 2 × 10^8^ sperm/mL.

### 2.3 Semen Process and Experimental Groups

Following dilution, the pooled semen was divided into nine equal portions. Each portion was supplemented with a different concentration of either VitB12 or VitB1. The control group did not receive any vitamin supplementation, while the experimental groups were supplemented with the following concentrations of VitB12 (mg/mL): 0.5 (B12‐0.5), 1 (B12‐1), 2 (B12‐2) and 4 (B12‐4); and VitB1 (mg/mL): 0.5 (B1‐0.5), 1 (B1‐1), 2 (B1‐2) and 4 (B1‐4). Both vitamins were purchased from Kimia Roshd Co., Iran. Stock solutions of each vitamin were prepared by dissolving the required amount in sterile phosphate‐buffered saline (PBS; pH 7.4) and stored at −20°C. The appropriate concentration for each experimental group was achieved by adding the vitamin stock solution to the semen samples. Each semen–vitamin mixture was thoroughly mixed to ensure uniform distribution of the supplements.

### 2.4 Cryopreservation Procedure

Cryopreservation was performed according to standard protocols for sperm freezing (Nateq et al. 2020). The diluted semen samples were loaded into 0.25 mL French semen straws using a sterile syringe, and the ends of the straws were sealed using a heat sealer. The semen straws were placed in an insulated container at 4°C for 90 min to equilibrate, allowing the sperm to adjust to the freezing conditions. After equilibration, the straws were exposed to vapour above liquid nitrogen for 10 min to initiate a controlled cooling process. This vapour exposure was followed by full immersion of the straws into liquid nitrogen at –196°C for long‐term storage. The liquid nitrogen tank used for storage was monitored regularly to ensure the temperature remained consistent. Semen was collected weekly for 5 weeks, with six replicates per collection. A total of 30 observations were made for each treatment group to ensure statistical reliability. After 1 week of storage, the straws were thawed in a water bath at 37°C for 30 s. Following thawing, sperm motility, viability and other quality parameters were assessed. Subsequently, spermatozoa were separated from the seminal plasma by centrifugation of the samples at 1800 × *g* for 10 min. The resulting supernatant was utilized to evaluate antioxidant activity in the seminal plasma.

### 2.5 Assessment of Sperm Quality Parameters

Sperm motility was assessed using computer‐assisted sperm analysis (CASA; Video Test‐Sperm 2.1, St. Petersburg, Russia). Following standard thawing procedures, sperm quality parameters from each replicate were evaluated across five microscopic fields. The integrity of the sperm membrane was examined using the hypo‐osmotic swelling test (HOST). For this test, a hypo‐osmotic solution was prepared with 9 g of fructose and 4.9 g of sodium citrate per litre of distilled water. A 5 µL semen sample was mixed with 95 µL of this solution and incubated at 37°C for 60 min. Post‐incubation, the samples were observed under a phase‐contrast microscope, and spermatozoa with swollen or coiled tails were recorded (Crisol et al. 2012).

Sperm viability was assessed using eosin–nigrosin staining. A smear was prepared by mixing 2 µL of semen with 10 µL of a staining solution containing 10 g of nigrosin, 1.7 g of eosin and 2.9 g of sodium citrate in 100 mL of distilled water. Live spermatozoa were identified by their ability to exclude the dye, whereas dead sperm cells appeared stained by eosin against a nigrosin background (magnification: 400×) (Jha et al. 2018).

Morphological abnormalities were evaluated by preparing semen smears stained with eosin–nigrosin. The staining solution consisted of 1% eosin B and 5% nigrosin in a 3% sodium citrate dihydrate solution. A drop of raw semen was mixed with a drop of the stain, and a fresh smear was prepared. A total of 200 spermatozoa per slide were examined at 1000× magnification using oil immersion, and the percentage of morphologically abnormal sperm, including defects in the head, midpiece and tail, was determined (Azubuike et al. 2017).

### 2.6 Measurement of Lipid Peroxidation

Malondialdehyde (MDA) concentration in diluted semen was determined as a marker of lipid peroxidation (LPO) using the thiobarbituric acid (TBA) assay. In this process, 1 mL of diluted semen was mixed with 1 mL of TBA solution, 1 mL of butylated hydroxytoluene (BHT) solution and 1 mL of ethylenediaminetetraacetic acid (EDTA). Proteins were precipitated by adding trichloroacetic acid (TCA) to the mixture, which was then centrifuged to separate the precipitate. Then, 1 mL of the supernatant was mixed with 1 mL of TBA and incubated in a boiling water bath for 10 min. After cooling, the absorbance of the solution was measured using a spectrophotometer (Amini et al. 2015; Daghigh Kia and Vatankhah 2019).

### 2.7 Measurement of TAC

The TAC in diluted semen was measured using the ferric reducing antioxidant power (FRAP) reagent, including tripyridyltriazine in HCl, FeCl_3_ and acetate buffer mixture in a 1:1:10 ratio. Then, 50 µL of the sample was added to 1.5 mL of the warm FRAP reagent and incubated at 37°C for 10 min. Absorbance was measured relative to a blank (Khadir et al. 2015).

### 2.8 Measurement of SOD

The activity of SOD in diluted semen was measured using the Ransod assay kit provided by Randox Laboratories (UK). This method generates superoxide radicals through the interaction of xanthine and xanthine oxidase. These radicals then react with 2‐(4‐iodophenyl)‐3‐(4‐nitrophenol)‐5‐phenyltetrazolium chloride (INT), resulting in the formation of a red formazan dye. To prepare the sample, the semen was diluted with a phosphate buffer. The SOD activity was quantified based on the extent of inhibition of the INT reduction, specifically achieving 50% inhibition.

### 2.9 Measurement of GPx

GPx activity diluted semen was evaluated using a spectrophotometric method. This approach is predicated on the role of GPx in catalysing the oxidation of reduced glutathione (GSH) by cumene hydroperoxide, with the involvement of glutathione reductase (GR) and NADPH. During this process, GR regenerates reduced GSH from its oxidized form, and NADPH is converted to NADP+ (N'Guessan et al. 2023).

### 2.10 Measurement of CAT

The activity of CAT was determined following the protocol described by Hu et al. (2011). For this assay, 0.3 mL of diluted semen was mixed with 1.7 mL of a substrate solution, which contained 65 µM H_2_O_2_ in 50 mM phosphate buffer. The mixture was incubated at 37.5°C for 60 s. To stop the reaction, 1.0 mL of 32.4 mM ammonium molybdate was added. The remaining H_2_O_2_ concentration was then assessed using a spectrophotometer, with a blank sample that included all components except the enzyme serving as a control.

### 2.11 Statistical Analysis

The differences in sperm quality parameters and antioxidant properties of diluted semen across the different treatment groups were analysed using one‐way analysis of variance (ANOVA), followed by Duncan's multiple range test for post hoc comparisons. Levene's test was performed to assess the homogeneity of variances across the groups. If homogeneity of variance was violated, Welch's ANOVA was applied as an alternative. The analysis was conducted using SPSS software (version 22). Pearson's correlation coefficient was calculated to evaluate the relationships between the parameters. A significance level of *p* ≤ 0.05 was considered statistically significant.

## 3 Results

### 3.1 Sperm Quality Parameters

The results indicated that the inclusion of varying concentrations of VitB12 and VitB1 in the diluent significantly impacted sperm quality parameters post‐freezing when compared to the control group (Table 1). Semen exposed to 0.5, 1, 2 and 4 mg/mL VitB12, along with lower levels of VitB1 (0.5 and 1 mg/mL), showed improvements in total and progressive motility, viability and plasma membrane integrity of spermatozoa relative to the control (*p* < 0.01). However, high concentrations of VitB1 (2 and 4 mg/mL) did not affect sperm characteristics compared to the control. The extender containing 1 mg/mL VitB12 and 0.5 or 1 mg/mL VitB1 was associated with lower sperm morphological defects compared to the control (*p* < 0.01).

**TABLE 1:** Effect of different levels of VitB12 and VitB1 (mg/mL of dilution) on the sperm quality parameters (%) after cryopreservation in rams.

Treatments	Overall motility	Progressive motility	Viability	Plasma membrane integrity	Abnormality
Control	50.12 ± 2.04^d^	42.25 ± 1.55^d^	55.75 ± 2.66^d^	51.50 ± 1.71^d^	7.75 ± 0.63^ab^
B12‐0.5	60.7 ± 2.28^c^	54.36 ± 1.68^c^	65.30 ± 2.20^c^	60.25 ± 1.03^c^	7.52 ± 0.65^abc^
B12‐1	73.66 ± 2.39^b^	65.20 ± 2.69^b^	77.44 ± 1.55^b^	71.20 ± 1.11^b^	4.26 ± 0.24^d^
B12‐2	58.74 ± 3.75^c^	56.03 ± 3.40^c^	65.20 ± 3.50^c^	58.52 ± 3.43^c^	7.68 ± 0.48^ab^
B12‐4	63.40 ± 2.20^c^	57.22 ± 2.86^c^	69.28 ± 2.21^c^	63.53 ± 2.75^c^	6.08 ± 1.08b^cd^
B1‐0.5	78.36 ± 2.47^ab^	72.50 ± 2.40^a^	84.27 ± 1.93^ab^	78.20 ± 2.03^a^	5.72 ± 0.49^cd^
B1‐1	83.70 ± 3.15^a^	78.11 ± 2.94^a^	86.02 ± 2.94^a^	78.54 ± 2.53^a^	5.26 ± 0.51^d^
B1‐2	50.03 ± 2.04^d^	42.25 ± 1.80^d^	56.75 ± 1.70^d^	51.24 ± 1.90^d^	9.12 ± 0.45^a^
B1‐4	45.20 ± 2.12^d^	40.17 ± 1.31^d^	52.00 ± 2.16^d^	45.63 ± 2.72^d^	9.34 ± 0.62^a^
SEM	2.42	2.28	2.10	2.02	0.32
*p* value	0.0001	0.0001	0.0001	0.0001	0.0001

*Note*: Means (±SE) with a different superscript alphabet in the same column differ significantly (*p* ≤ 0.05).

Abbreviation: SEM, standard error mean.

### 3.2 LPO and Antioxidant Capacity

**FIGURE 1:**
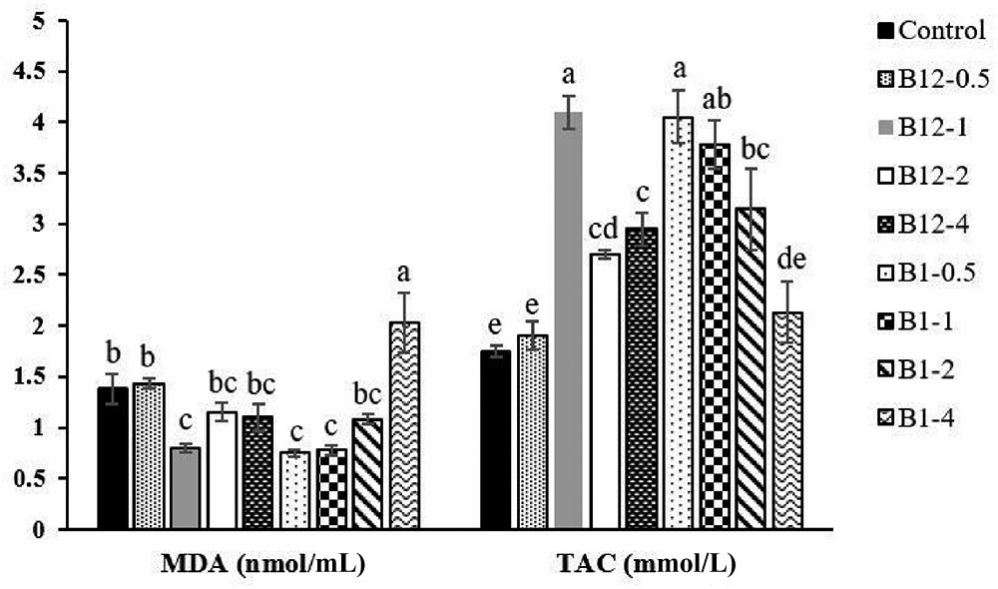
Effect of different levels of VitB12 and VitB1 (mg/mL of diluent) on the malondialdehyde (MDA) and total antioxidant capacity (TAC) levels in diluted semen after cryopreservation. Different letters above the bars for each parameter indicate significant differences among groups (*p *< 0.01).

As illustrated in Figure 1, the level of MDA (nmol/mL) was significantly reduced (*p* < 0.05) in the B12‐1 (0.80 ± 0.04), B1‐0.5 (0.75 ± 0.03) and B1‐1 (0.78 ± 0.05) groups compared to the control (1.38 ± 0.15). The highest concentration of VitB1 (B1‐4) resulted in the highest MDA level (2.03 ± 0.29) among the treatments (*p* < 0.05). In addition, TAC (mmol/mL) was significantly higher in the B12‐1 (4.10 ± 0.16), B12‐2 (2.70 ± 0.04), B12‐4 (2.95 ± 0.16), B1‐0.5 (4.05 ± 0.26), B1‐1 (3.78 ± 0.24) and B1‐2 (3.15 ± 0.40) groups compared to the control group (1.75 ± 0.06).

### 3.3 Antioxidant Enzymes

SOD activity in diluted semen was unaffected by the different levels of VitB12 and VitB1 in the diluents. However, the highest GPx activity (U/L) was observed in the B12‐1 (80.50 ± 4.41) and B1‐1 (81.14 ± 4.20) groups, significantly surpassing the control (20.25 ± 2.39) and other treatments (*p* < 0.01). The control group exhibited the lowest GPx activity among all experimental groups (*p* < 0.01). In addition, CAT activity was significantly elevated in the B12‐1 (7 ± 0.46), B12‐2 (6.18 ± 0.31), B1‐0.5 (6 ± 0.46), B1‐1 (7.25 ± 0.32) and B1‐2 (6.75 ± 0.10) groups compared to the control (4.20 ± 0.39) (*p* < 0.01) (Table 2).

**TABLE 2:** Effect of different levels of VitB12 and VitB1 (mg/mL of dilution) in diluent on the antioxidant enzymes of frozen‐thawed semen in rams.

	Antioxidant enzymes		
Treatments	SOD (U/mL)	GPx (U/L)	CAT (U/mL)
Control	66.67 ± 7.69	20.25 ± 2.39^e^	4.20 ± 0.39^c^
B12‐0.5	73.75 ± 4.27	34.05 ± 2.94^d^	4.68 ± 0.26^c^
B12‐1	81.00 ± 3.19	80.50 ± 4.41^a^	7.00 ± 0.46^ab^
B12‐2	80.00 ± 5.40	52.53 ± 4.60^c^	6.18 ± 0.31^b^
B12‐4	71.25 ± 4.73	49.25 ± 4.71^c^	3.83 ± 0.12^c^
B1‐0.5	70.50 ± 6.85	65.28 ± 2.87^b^	6.00 ± 0.46^b^
B1‐1	72.50 ± 3.23	81.14 ± 4.20^a^	7.25 ± 0.32^a^
B1‐2	70.50 ± 4.43	49.58 ± 2.10^c^	6.75 ± 0.10^ab^
B1‐4	65.51 ± 5.87	43.68 ± 1.57^cd^	4.58 ± 0.22^c^
SEM	1.72	3.36	0.23
*p* value	0.73	0.0001	0.0001

*Note*: Means (±SE) with a different superscript alphabet in the same column differ significantly (*p* ≤ 0.05).

Abbreviations: CAT, catalase; GPx, glutathione peroxidase; SEM, standard error mean; SOD, superoxide dismutase.

### 3.4 Correlation Between Antioxidant Activity and Sperm Quality

**FIGURE 2:**
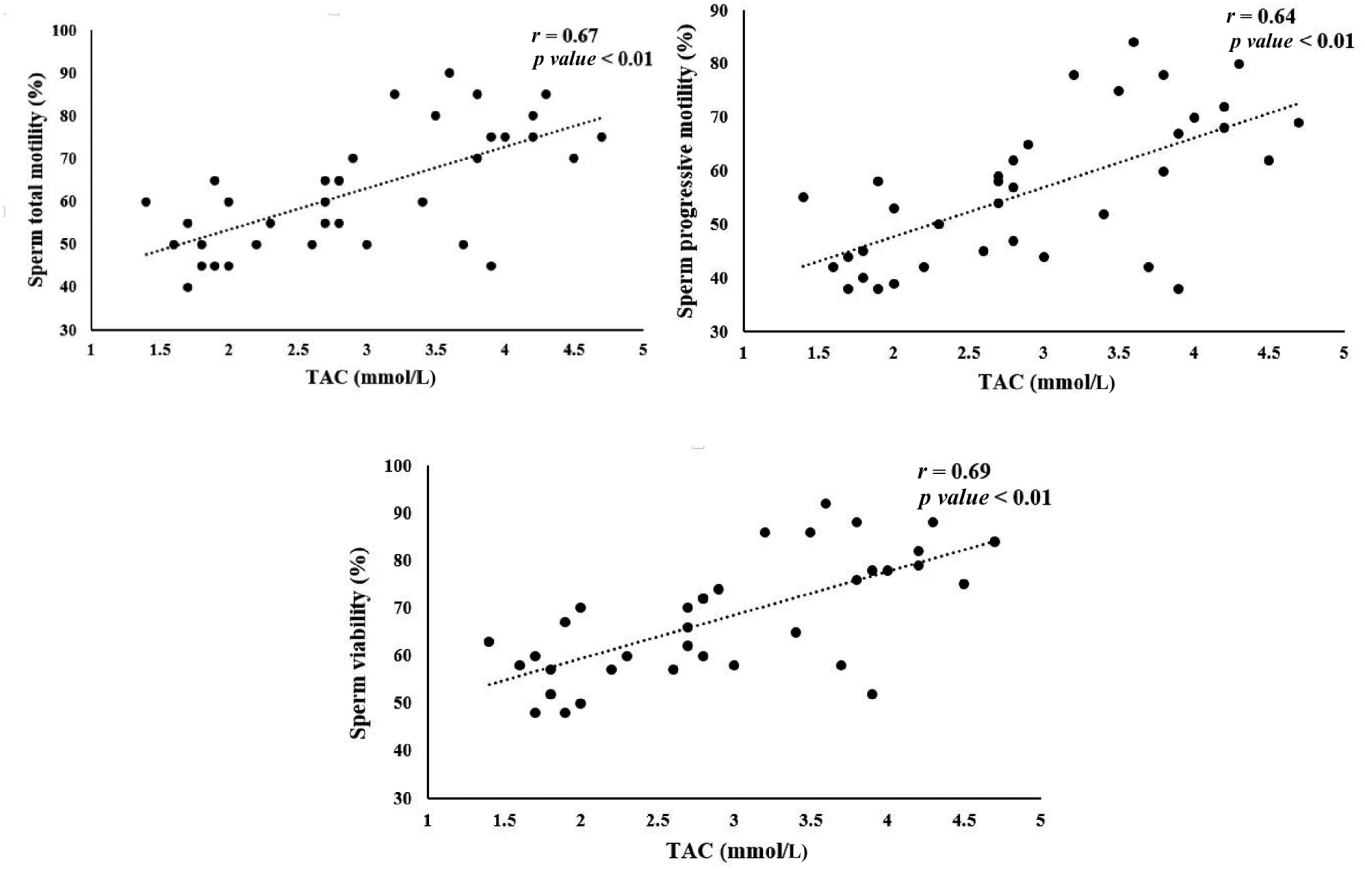
Significant correlations between sperm quality parameters and diluted semen TAC activity in rams.

**FIGURE 3:**
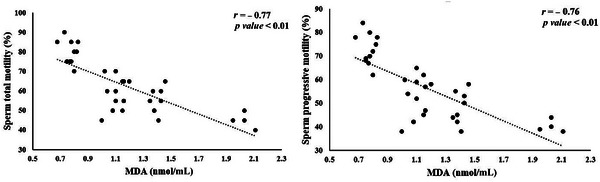
Significant correlations between sperm quality parameters and diluted semen MDA level in rams.

**FIGURE 4:**
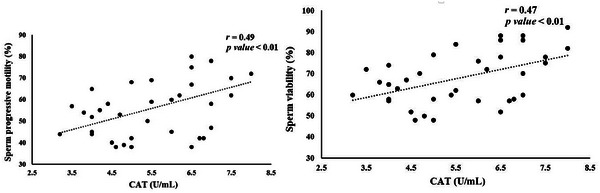
Significant correlations between sperm quality parameters and diluted semen CAT activity in rams.

**FIGURE 5:**
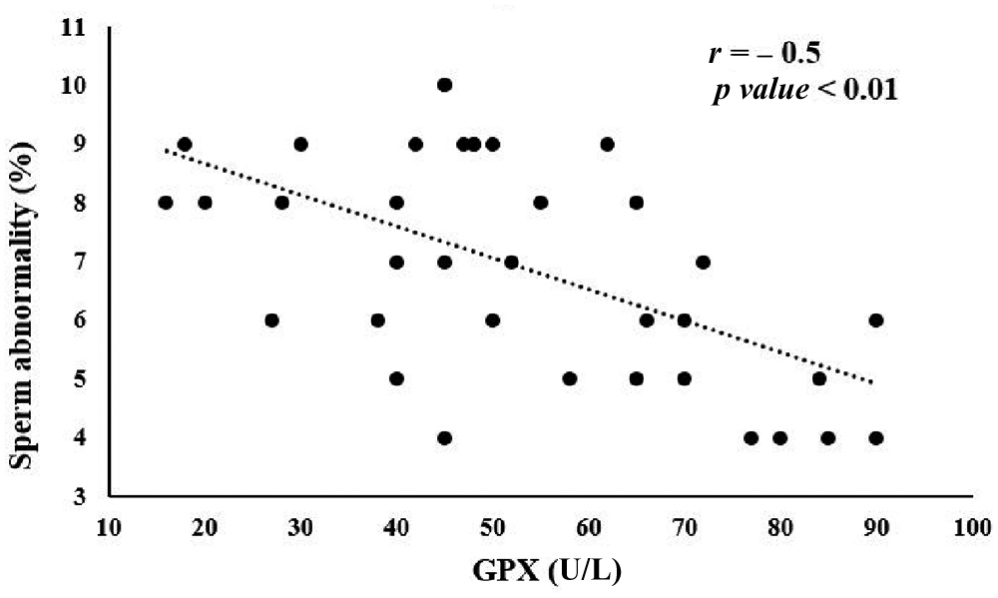
Significant correlation between sperm abnormality percent and diluted semen GPx activity in rams.

Significant correlations between seminal plasma antioxidant status and sperm quality parameters are illustrated in Figures 2, 3, 4, 5. TAC exhibited a notable positive correlation (*p* < 0.01) with total motility (*r* = 0.67), progressive motility (*r* = 0.64) and sperm viability (*r* = 0.69) (Figure 2). However, TAC did not show significant correlations with sperm plasma membrane integrity or normal sperm morphology. As shown in Figure 3, a significant negative correlation (*p <* 0.01) was found between diluted semen MDA levels and both total motility (*r* = −0.77) and progressive motility (*r* = −0.76). No significant relationship was observed between MDA levels and other sperm parameters. CAT activity in diluted semen was significantly correlated (*p* < 0.01) with progressive motility (*r* = 0.49) and sperm viability (*r* = 0.47) (Figure 4). However, CAT activity did not correlate significantly with total motility, plasma membrane integrity or sperm morphology. A significant negative correlation (*p* < 0.01, *r* = −0.5), was found between GPx activity in diluted semen and the percentage of sperm abnormalities, although no significant correlation was observed with other sperm quality parameters (Figure 5). The correlation between SOD activity and sperm quality parameters was not significant.

## 4 Discussion

Research into sperm phospholipid peroxidation indicates that this process can damage sperm membranes, leading to reduced motility and compromised membrane integrity (Shiva et al. 2011). This study investigated how varying concentrations of VitB12 and VitB1 in semen extenders influence sperm quality and antioxidant activity in diluted semen following freeze‐thaw procedures in Arabi rams. The findings revealed that adding VitB12 at concentrations of 0.5, 1, 2 and 4 mg/mL to the cryoprotective medium significantly improved sperm motility, viability and membrane integrity after thawing. Notably, only the 1 mg/mL VitB12 concentration effectively reduced sperm morphological abnormalities. Although antioxidant supplementation generally benefits sperm morphology, results can vary; some studies report no change or even deterioration in sperm quality (Noegroho et al. 2022). Prior studies have documented that VitB12 in extenders enhances several sperm quality parameters, including motility, viability, membrane integrity and morphology across different breeds, such as Ghezel × Baluchi and Ghezel × Arkharmerino rams (Asadpour et al. 2012) and Dallagh rams (Hamedani et al. 2013), during liquid semen storage at 5°C. Ahmed et al. (2021) observed that 4 and 5 mg/mL VitB12 significantly improved progressive motility in buffalo bull semen extenders. Similarly, Suwimonteerabutr et al. (2024) found that 0.04% of VitB12 enhanced sperm quality in chilled semen extenders for roosters. For bovine semen, 2.50 mg/mL of VitB12 improved motility and membrane integrity, whereas higher doses (3.75 mg/mL) were found to be detrimental (Hu et al. 2011). Mizera et al. (2019) identified 2.50 mg/mL as the optimal concentration for bull semen extenders, with no additional benefits observed at 5 mg/mL. Research in humans has shown mixed outcomes: some studies found a positive association between VitB12 levels and sperm motility and structure (Ray et al. 2020), while others noted no significant correlation between sperm parameters and serum VitB12 levels (Boushaba et al. 2023), though seminal VitB12 content was linked with sperm parameters (Boxmeer et al. 2009). VitB12 supplementation has been noted to enhance motility and viability and reduce DNA fragmentation in human spermatozoa, suggesting that a concentration of 2 mg/mL VitB12 in cryopreservation media may help mitigate sperm damage (Hosseinabadi et al. 2020). VitB12 deficiency in male rats has been associated with decreased motility and increased sperm abnormalities (Watanabe et al. 2003). The benefits of VitB12 on semen quality might be attributed to increased nitric oxide production, reduced ROS accumulation, enhanced energy production and protection against oxidative stress through modulation of cytokines and growth factors, as well as decreased homocysteine‐induced oxidative stress (Alzoubi et al. 2014; Banihani 2017; Hosseinabadi et al. 2020).

Research into the effects of thiamine on male reproductive health, especially in farm animals, is limited. This study is among the first to explore how VitB1, in conjunction with VitB12, influences reproductive performance in rams. The results revealed that lower concentrations of VitB1 (0.5 and 1 mg/mL) positively impacted sperm quality in Arabi rams. Conversely, higher concentrations (2 and 4 mg/mL) did not improve sperm characteristics after freezing and thawing. Thiamine is essential as a cofactor in oxidative energy metabolism, and its presence in semen has been positively linked to sperm concentration (Gangolf et al. 2010).

Overproduction of ROS in diluted semen is associated with reduced sperm fertilization ability, impaired metabolism and negative effects on sperm motility and morphology (Lopes et al. 2021). In our study, treatment with 1 mg/mL VitB12 and 0.5–1 mg/mL VitB1 led to a significant decrease in MDA levels in diluted semen compared to the control group. However, an increase in MDA levels was observed with 4 mg/mL VitB1, suggesting diminished antioxidant activity relative to other treatments. TAC was notably higher in the groups treated with 1–4 mg/mL VitB12 and 0.5–2 mg/mL VitB1 compared to the control. It is important to note that excessive antioxidant supplementation can sometimes act as a pro‐oxidant, potentially leading to increased oxidative stress and disruption in the balance between ROS production and neutralization (Poljsak et al. 2013; Suwimonteerabutr et al. 2024). Previous studies have demonstrated that adding 5 mg/mL VitB12 to buffalo bull seminal extenders decreased ROS activity in samples relative to controls (Ahmed et al. 2021). In addition, individuals with VitB12 deficiency were found to have lower TAC and elevated MDA levels compared to those without deficiency (Misra et al. 2017). TAC measures the overall antioxidant capability in biological fluids and is vital for evaluating redox status (Rodrigo et al. 2013). VitB12's antioxidant properties include scavenging ROS, maintaining GSH levels and reducing oxidative stress induced by homocysteine (Siddiqua et al. 2024). Deficiency in VitB12 has been linked to increased cellular H_2_O_2_ and nitric oxide (NO), decreased activities of antioxidant enzymes like SOD and CAT and significant oxidative stress (Bito et al. 2017). Nonetheless, Güney et al. (2015) reported no significant differences in oxidative stress markers between VitB12‐deficient patients and healthy controls.

Our study revealed a significant positive relationship between the TAC in semen and sperm parameters, including total motility, progressive motility and viability. In contrast, MDA levels were negatively correlated with these sperm quality indicators. The interplay between antioxidant capacity and oxidative damage in sperm is closely linked to TAC and MDA levels in semen (Tavilani, Doosti, and Saeidi 2005). Fazeli and Salimi (2016) found that men with idiopathic infertility exhibited lower TAC and higher MDA levels, which were associated with diminished sperm motility and morphology. However, in rams, no significant correlation was observed between TAC, MDA and normal sperm morphology. Conversely, another study reported a positive correlation between TAC and all semen parameters in men (Pahune et al. 2013).

Elevated levels of ROS are known to negatively impact male fertility by impairing sperm function. Adequate antioxidant levels in semen are essential for shielding sperm from ROS‐induced damage, with reduced TAC frequently associated with male infertility (Mahfouz et al. 2009). Our study found a notable correlation between CAT activity in diluted semen and sperm progressive motility and viability. CAT is crucial for converting H_2_O_2_ into water and oxygen, and its supplementation has been reported to alleviate the negative effects of extended semen storage in sheep (Hakim Abed Bresm and Mohammed Hassan Habeeb 2023). Although Khosrowbeygi et al. (2004) found a significant link between CAT activity and sperm motility and morphology in men, we did not observe similar associations in rams.

Our study revealed a significant negative correlation between GPx activity and sperm morphological abnormalities. This contrasts with the findings by Rajoriya et al. (2013), who did not observe significant correlations between sperm viability and CAT activity in frozen‐thawed bull semen. GPx helps protect sperm DNA from oxidative damage by reducing peroxides through electron transfer from GSH. Although increased GPx activity has been observed in normozoospermic individuals compared to those with sperm dysfunctions, its relationship with sperm quality parameters can vary (Chen et al. 2003; Kowalczyk 2022). Immature and abnormal sperm cells, which often have impaired mitochondrial function, are significant contributors to ROS in semen (Ribeiro et al. 2021). While our study did not find significant correlations between sperm motility and GPx activity in rams, other studies in men have reported such associations (Crisol et al. 2012).

SOD, produced by various reproductive tissues and cells, plays a role in maintaining sperm motility (Qamar et al. 2023). In our study, we did not find a significant correlation between SOD activity and sperm quality parameters in rams. The association between SOD activity in semen and sperm quality is still under debate. While some studies report positive correlations, particularly in infertile men (Gavella et al. 1996; Kurpisz et al. 1996; Murawski et al. 2007), others have found no significant relationships (Hsieh et al. 2002; Khosrowbeygi et al. 2004; Patricio et al. 2016; Zalata et al. 1995; Zini et al. 1993).

B‐group vitamins can display both antioxidant and pro‐oxidant properties depending on the experimental settings (Higashi‐Okai et al. 2006). While the use of antioxidants to enhance male fertility has been extensively investigated, the ideal treatment protocols and effective physiological concentrations are still not well‐defined. Additional research is required to determine the most effective antioxidant combinations for addressing male infertility, taking into account various environmental factors (Dimitriadis et al. 2023).

## 5 Conclusion

This study found that lower concentrations of vitamins B12 and B1 had a beneficial effect on the quality of ram semen. Supplementing the cryoprotective media with 1 mg/mL VitB12 and 0.5 mg/mL VitB1 improved various sperm quality parameters and provided enhanced protection against oxidative stress. Significant correlations were identified between sperm quality and several antioxidant factors in semen, including TAC, MDA levels and specific antioxidant enzymes. These results indicate that the observed improvement in sperm quality during freezing and thawing can be attributed to the increased antioxidant protection offered by vitamins B1 and B12.

## Author Contributions

**Saleh Tabatabaei Vakili**: methodology, formal analysis, supervision, writing–review and editing. **Roghayeh Zeidi**: data curation, investigation, writing–original draft. **Robab Nabhani**: methodology, investigation, resources.

## Ethics Statement

The Animal Ethics Committee, Department of Animal Science, Agricultural Sciences and Natural Resources University of Khuzestan agreed upon all animal care and procedures according to US National Research Council's guidelines, project code: 1.411.182.

## Conflicts of Interest

The authors declare no conflicts of interest.

### Peer Review

The peer review history for this article is available at https://publons.com/publon/10.1002/vms3.70241.

## Data Availability

The data supporting this study's findings are available on request from the corresponding author.
